# Man and Insects

**Published:** 1883-07

**Authors:** Grant Allen


### MAN AND INSECTS.

THE only nerves (worth mentioning) in the human body which are not under the control of the brain, are those of the heart and other internal organs ; and over these parts, as everybody knows, we have not any voluntary power. But all our limbs and muscles are moved in accordance with impulses sent down from the brain, so that, for example, when I have made up my mind to send a telegram to a friend, my legs take me duly to the telegraph office, my hand writes the proper message, and my tongue undertakes the necessary arrangements with the clerk. But in the insect's body there is no such regular subordination of all the parts composing the nervous system to a single central organ or head office. The largest knot of nerve-matter, it is true, is generally to be found in the neighborhood of the sense-organs, and it receives direct nerve-bundles from the eyes, antennæ, mouth, and other chief adjacent parts ; but the wings and legs are moved by separate knots of nerve cells connected by a sort of spinal cord with the head, but capable of acting quite independently on their own account.

Thus, if we cut off a wasp's head and stick it on a needle in front of some sugar and water, the mouth will greedily begin to eat the sweet syrup, apparently unconscious of the fact that it has lost its stomach, and that the food is quietly dropping out of the gullet at the other end as fast as it is swallowed. So, too, if we decapitate that queer Mediterranean insect, the praying mantis, the headless body will still stand catching flies with its outstretched arms, and fumbling about for its mouth when it has caught one, evidently much surprised to find that its head is unaccountably missing. In fact, whatever may be the case with man, the insect, at least, is really a conscious automaton. It sees or smells food, and it is at once impelled by its nervous constitution to eat it. It receives a sense impression from the bright hue of a flower, and it is irresistibly attracted toward it, as the moth is to the candle. It has no power of deliberation, no ability even to move its own limbs in unaccustomed manners. Its whole life is governed for it by its fixed nervous constitution, and by the stimulations it receives from outside. And so, though the world probably appears much the same to the beetle as to us, the nature of its life is different. It acts like a piece of clock-work mechanism, wound up to perform a certain number of fixed movements, and incapable of ever going beyond the narrow circle for which it is designed.—*Grant Allen in Knowledge.*

---

SUFFERING is the plow that turns up the field of the soul, in whose deep furrows the all-wise husbandman scatters His heavenly seed.

